# MTHFD1 regulates the NADPH redox homeostasis in MYCN-amplified neuroblastoma

**DOI:** 10.1038/s41419-024-06490-3

**Published:** 2024-02-09

**Authors:** Jinqiu Guan, Mengzhen Li, Yi Wang, Yu Zhang, Yi Que, Suying Lu, Juan Wang, Jia Zhu, Junting Huang, Zijun Zhen, Feifei Sun, Mengjia Song, Yizhuo Zhang

**Affiliations:** 1 Department of Pediatric Oncology, Sun Yat-sen University Cancer Center, Guangzhou, China; 2 State Key Laboratory of Oncology in South China, Guangdong Provincial Clinical Research Center for Cancer, Collaborative Innovation Center for Cancer Medicine, Sun Yat-sen University Cancer Center, Guangzhou, China

**Keywords:** Paediatric cancer, Oncogenes

## Abstract

MYCN amplification is an independent poor prognostic factor in patients with high-risk neuroblastoma (NB). Further exploring the molecular regulatory mechanisms in MYCN-amplified NB will help to develop novel therapy targets. In this study, methylenetetrahydrofolate dehydrogenase 1 (MTHFD1) was identified as the differentially expressed gene (DEG) highly expressed in MYCN-amplified NB, and it showed a positive correlation with MYCN and was associated with a poor prognosis of NB patients. Knockdown of MTHFD1 inhibited proliferation and migration, and induced apoptosis of NB cells in vitro. Mouse model experiments validated the tumorigenic effect of MTHFD1 in NB in vivo. In terms of the mechanism, ChIP-qPCR and dual-luciferase reporter assays demonstrated that MTHFD1 was directly activated by MYCN at the transcriptional level. As an important enzyme in the folic acid metabolism pathway, MTHFD1 maintained the NADPH redox homeostasis in MYCN-amplified NB. Knockdown of MTHFD1 reduced cellular NADPH/NADP^+^ and GSH/GSSG ratios, increased cellular reactive oxygen species (ROS) and triggered the apoptosis of NB cells. Moreover, genetic knockdown of MTHFD1 or application of the anti-folic acid metabolism drug methotrexate (MTX) potentiated the anti-tumor effect of JQ1 both in vitro and in vivo. Taken together, MTHFD1 as an oncogene is a potential therapeutic target for MYCN-amplified NB. The combination of MTX with JQ1 is of important clinical translational significance for the treatment of patients with MYCN-amplified NB.

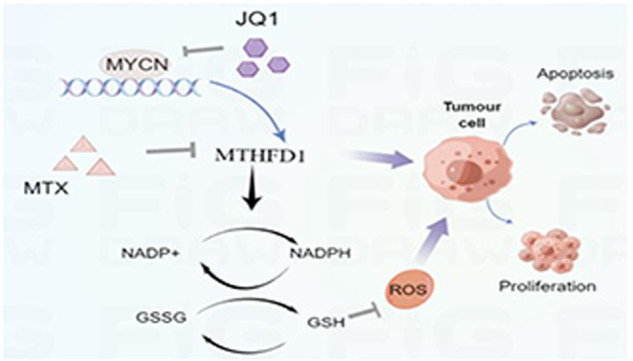

## Introduction

Neuroblastoma (NB) generally originating from the sympathetic nervous system or adrenal glands is the most common malignancy in infants, accounting for about 6% of all cancers in childhood. MYCN amplification, which occurs in 20–30% of patients with NB, is an important risk stratification factor and strongly correlated with a poor prognosis in high-risk NB [1, 2]. Due to the presence of an α-helix structure on the surface, it is difficult for drug inhibitors to target MYCN directly. Small molecule JQ1 is a BET inhibitor that disrupts the correlation of BET proteins with transcription factors, suppresses the expression of oncogenes, and eventually causes the cessation of tumor cell growth. JQ1 can down-regulate the expression of MYCN in NB, but its effect seems unsatisfactory when used alone [3, 4]. Therefore, it is of great significance to further explore the molecular mechanism of MYCN-amplified NB for finding potential novel therapy targets.

Accumulating evidence has indicated the importance of metabolic abnormality in tumor progression [5–8]. The process of one-carbon metabolism, which plays an irreplaceable role in tumorigenesis, mainly consists of the folate cycle and methionine cycle, through which purine, adenosine, and other metabolites are generated. As a crucial part of carbon metabolism, the folate cycle can provide much energy for tumor cell proliferation [9], which occurs in the cytoplasm and mitochondria. Methylenetetrahydrofolate dehydrogenase 1 (MTHFD1), as a key enzyme in the folate cycle, existing in the cytoplasm, contains three functional groups with different catalytic activities, which is closely related to NADPH production and adenosine purine nucleotide metabolism, including dehydrogenase, cyclohydrolase, and tetrahydrofolate synthetase [10–12]. MTHFD1 is upregulated in multiple tumors such as cholangiocarcinoma, colorectal cancer, melanoma, and hematologic malignancies [13–15], which is related to tumor apoptosis, proliferation, migration and drug resistance. However, the role of MTHFD1 in NB and its molecular mechanisms remain obscure. This study aimed to explore the clinical significance, biological function, and regulatory mechanisms of MTHFD1 in MYCN-amplified NB. Meanwhile, the effect of MTHFD1 knockdown or anti-folic acid metabolism drug methotrexate (MTX) combined with the BRD4 inhibitor JQ1 was also evaluated, aiming to identify the potential of MTHFD1 as a therapy target for NB, and to reveal the importance of targeted inhibition of folic acid metabolism pathway for NB therapy.

## Materials and methods

### Cell lines and clinical samples

Cell lines used in this study were purchased from the COBIOER BIOSCIENCES (Nanjing, China). Cell lines had been authenticated by STR profiling and free from mycoplasma. Human NB cell lines SK-N-BE(2) and SHSY-5Y were cultured in MEM/F12 (1:1) medium; human NB cell lines IMR32 and SK-N-SH were cultured in MEM medium; and human NB cell lines SK-N-AS were cultured in DMEM medium, with all mediums supplemented with 10% fetal bovine serum (FBS). All cells were cultured in a humidified incubator with 5% CO_2_ and maintained at 37 °C. Human tissue samples were obtained from Sun Yat-sen University Cancer Center under protocols approved by the Institutional Review Board. All patients were ≤18 years old with initial diagnosis as NB. NB patients >18 years old or with incomplete clinical data were excluded out. Written informed consent was obtained from each tissue donor and all procedures were conducted following the medical ethical guidelines. Demographics of NB patients from our center were summarized in Supplementary Table 1.

### Immunohistochemistry (IHC) assay

The IHC assay was performed on 57 NB tissue samples obtained from our center [16]. Anti-MTHFD1 mouse mAb was used in this assay. The staining results were evaluated based on the intensity and the proportion of positive-stained tumor cells. The intensity was scored as follows: 0 - negative; 1 - weak; 2 - moderate; 3 - strong. The proportion of positive cells was scored as follows: 0–less than 25%; 1–25% to 50%; 2–50% to 75%; 3–75% to 100%. The composite staining score (the product of the above two scores) of 0–4 was considered a low expression, and that of 5–9 was considered a high expression.

### Transfection, lentiviral transduction, and RNAi

SK-N-BE(2) cell lines stably expressing MTHFD1 shRNA were generated through lentiviral transduction followed by puromycin selection. To obtain the shRNA-expressing virus, shRNA vectors were co-transfected with the lentivirus packaging plasmids into HEK293T cells using Lipofectamine 3000 (Invitrogen, USA). Fresh media were added after 6-8 h, and viral supernatants were collected at 48 h. Target cells were infected with viral supernatant (diluted at 1:1 with fresh media; 8 μg/mL polybrene), added with fresh media 24 h later and selected with 1 μg/mL puromycin. SK-N-AS cells overexpressing MYCN were generated using the same packaging system and MYCN plasmid. For MTHFD1 and MYCN knockdown, siRNA targeting each gene (RiboBio, Guangzhou, China) was transfected into SK-N-BE(2) or IMR32 cells using Lipofectamine RNAiMAX (Invitrogen, USA).

### Realtime quantitative polymerase chain reaction (RT-qPCR)

Total RNA was isolated with TRIzol according to the manufacturer’s instruction, and subjected to RT-qPCR using SYBR PrimeScript RT Master Mix, with β-actin as an internal control. The primer sequences were shown in Supplementary Table 2.

### Western blotting (WB)

Cell lysates were prepared, and proteins were separated by SDS-PAGE and transferred onto a PVDF membrane. Then the membrane was blocked in 5% skim milk or 5% BSA in Tris-buffered saline (TBS, 10 mM Tris, 10 mM NaCl) and incubated overnight with primary antibodies against β-actin (Proteintech, Wuhan, China, 66009), MYCN (Santa Cruz Biotechnology, USA, sc-53993), MTHFD1 (Proteintech, Wuhan, China, 10794), and GAPDH (Proteintech, Wuhan, China, 60004). The next day, it was incubated again with goat anti-rabbit immunoglobulin G (FUDE, Hangzhou, China, FDR007) or goat anti-mouse immunoglobulin G (FUDE, Hangzhou, China, FDM007) secondary antibodies.

### Cell proliferation and IC50 assay

The cell proliferation rate was measured using Cell Counting Kit 8 (CCK-8, Dojindo, Shanghai, China). Specifically, cells were seeded at a density of 2000 cells per well in a 96-well plate. 24 h later, the number of viable cells was detected with Cell Titer Glo reagent daily. In IC50 assay, cells were seeded at a density of 2000 cells per well in a 96-well plate for 24 h, and then treated with JQ1 for 96 h. The number of viable cells was detected with Cell Titer Glo reagent.

### Colony formation assay

In colony formation assay, 5 × 10^4^ cells were seeded in a 6-well plate and incubated with indicated compounds for 10–14 days until the obvious colony was formed. Then, the plate was gently washed and stained with crystal violet for colony visualization.

### Cell apoptosis analysis

In the cell apoptosis assay, Annexin V-FITC/PI Apoptosis Detection Kit (4 A Biotech, Beijing, China) was used according to the manufacturer’s instructions. Briefly, cells were collected, washed, resuspended in binding buffer, sequentially stained with Annexin V-FITC and PI, and immediately analyzed by flow cytometry (SP6800, Sony, Japan).

### Detection of NADPH/NADP^+^ and GSH/GSSG ratios, and ROS content

The intracellular NADPH/NADP^+^ and GSH/GSSG ratios were measured with NADPH/NADP^+^ Assay Kit (Promega, USA, #G9081) and GSH/GSSG Assay Kit (Beyotime, Shanghai, China, S0053), respectively, according to the manufacturer’s instructions. The levels of cellular ROS were determined by flow cytometry using DCFH-DA (Beyotime, Shanghai, China, S0033s) as fluorescent probes following the manufacturer’s protocol.

### ChIP assay

Cells were fixed with 1% formalin and sonicated. Then sheared DNA was incubated with antibodies against MYCN (Santa Cruz Biotechnology, sc-53993) or IgG control (Santa Cruz Biotechnology, USA, sc-2027). DNA-protein-antibody complexes were incubated with Protein A Agarose/Salmon Sperm DNA Beads (Merck, Germany, CS204457). The beads were washed sequentially with gradient salt buffer and eluted in 1% SDS/NaHCO_3_. Finally, ChIP DNA was analyzed by qPCR.

### Luciferase assay

Cells were plated in a 96-well plate before transfection. Empty pGL3 luciferase vector, pGL3 expressing MTHFD1 or pGL3 MTHFD1 mutant was transiently co-transfected into HEK293T cells, using a control Renilla luciferase plasmid (pRL-TK). The test plasmid: control plasmid ratio was 50:1. Luciferase activities were measured 48 h later using Dual-Luciferase Reporter Gene Assay Kit (Vazyme, Nanjing, China, DL101-01). Firefly luciferase activities were normalized to the value of Renilla luciferase control and described by the average of triplicates.

### Animal experiments

Female NCG mice at 3 to 4-week-old were purchased from GemPharmatech Co., Ltd. (Jiangsu, China). All animal experiments were conducted in accordance with animal protocols approved by the Institutional Animal Care and Use Committee (IACUC) of Sun Yat-sen University. In MTHFD1 knockdown xenograft experiment, 1 × 10^7^ SK-N-BE(2) cells with stable MTHFD1 knockdown were implanted subcutaneously into the mice in the flank, with 6 mice for each group. Then subcutaneous tumors were harvested for ex vivo imaging followed by H&E or IHC staining for histological analysis. For the combined administration model, the mice were implanted with 1 × 10^7^ wild-type SK-N-BE(2) cells. After about 2 weeks, the tumor could be touched. Then the mice with tumors were randomly divided in to 4 groups, with 6 mice for each group. When the tumor volume reached about 100 mm^3^, drug administration was conducted. For the combination of shMTFHD1 and JQ1, JQ1 was intraperitoneally injected at 50 mg/kg/day for 14 consecutive days. For the combination of MTX and JQ1, JQ1 and MTX were intraperitoneally injected at 50 mg/kg/day and 20 mg/kg/day, respectively, for 14 consecutive days. Tumor volumes were measured using an electronic caliper and calculated using the following formula: tumor volume = length (mm) × width (mm) × width (mm) × 0.5. The tumor length reaching 2 cm in some mice could be regarded as the endpoint. Investigators were not blinded in this animal experiment.

### Bioinformatics analysis

The TARGET database of NB was searched (https://ocg.cancer.gov/programs/target), and the RNA sequencing data and basic clinical information of patients were included. Patients were divided into two groups: MYCN amplification group and MYCN non-amplification group. The limma package was used to screen the DEG with log |FC| >0.378, and an adjusted *P* < 0.001 was defined as the threshold of screening. Then R language was used to perform the KEGG (Kyoto Encyclopedia of Genes and Genomes) enrichment analysis, Spearman correlation analysis and Kaplan-Meier survival prognosis analysis.

### Statistical analysis

All statistical analyses of experiment results were performed using GraphPad Prism 8.0 software and repeated at least 3 times. The continuous results of statistical data were expressed as mean ± SD, with *P* < 0.05 considered as statistically significant. The variance was assumed as similar between compared groups with normal distribution. Comparisons of variables between two groups were performed using a two-tailed Student’s *t* test; comparisons of variables between multiple groups were performed using one-way ANOVA analysis followed by Tukey’s post hoc test; comparisons of the constituent ratios between MTHFD1 high-expression and MTHFD1 low-expression groups were performed using Chi-square test (*χ*^2^ test); Kaplan–Meier survival analysis was performed using log-rank test.

## Results

### MTHFD1 was upregulated in MYCN-amplified NB and correlated with the poor prognosis of NB patients

**Fig. 1:**
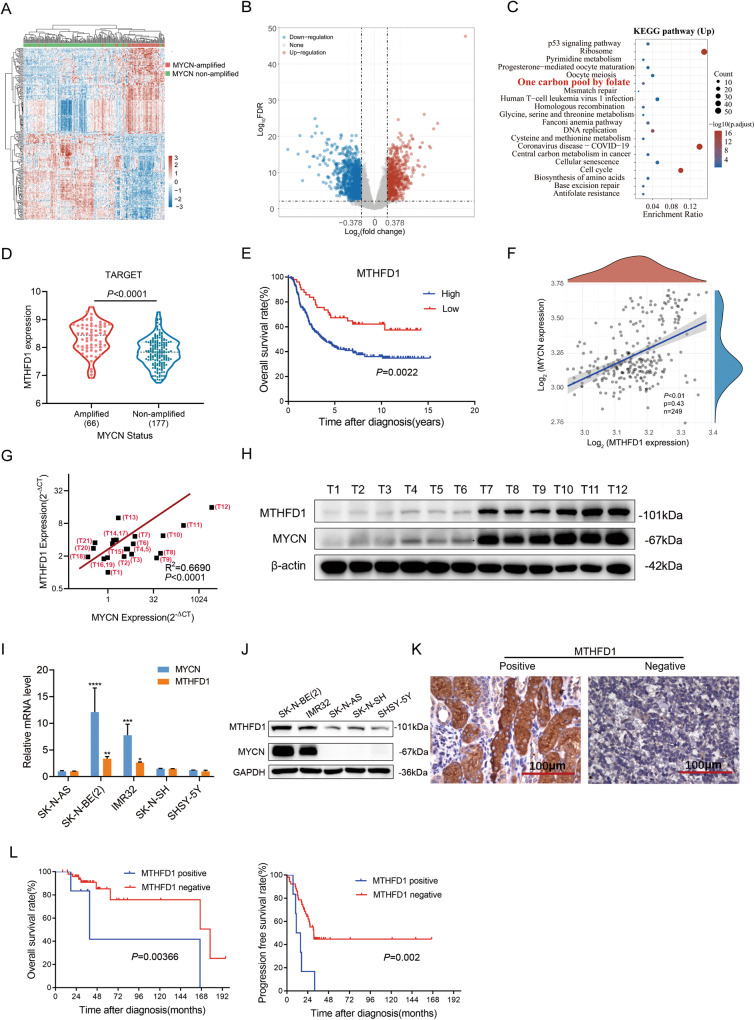
MTHFD1 was upregulated in MYCN-amplified NB and correlated with the poor prognosis of NB patients. **A** Heatmap revealed the DEGs between MYCN amplified and MYCN non-amplified NB patients. **B** Volcano plot showed DEGs between MYCN amplified and MYCN non-amplified NB patients. **C** KEGG pathway enrichment analysis of DEGs between MYCN amplified and MYCN non-amplified NB. **D** MTHFD1 expression level in NB patients with MYCN amplification and MYCN-non amplification based on the TARGET database. **E** Kaplan–Meier analysis of overall survival for NB patients with low vs high expression of MTHFD1 based on the TARGET database. **F** Correlation of MTHFD1 and MYCN by Spearman correlation analysis in 249 NB patients based on the TARGET database. **G** Scatterplots of MTHFD1 vs MYCN mRNA expression in NB tissues analyzed by RT-qPCR at our center. β-actin was used as the loading control. **H** WB analysis of MYCN and MTHFD1 in NB tissues with or without MYCN amplification. The original full-length western blots were provided as Supplementary Fig. 2. **I** RT-qPCR analysis of MTHFD1 and MYCN mRNA expression in MYCN amplified (SK-N-BE(2) and IMR32) and MYCN non-amplified NB cell lines (SK-N-AS, SHSY-5Y and SK-N-SH). β-actin was used as loading control. The mRNA levels in SK-N-AS being designated as 1.0. **J** WB analysis of MTHFD1 and MYCN protein expression in MYCN amplified and MYCN non-amplified NB cell lines. The original full length western blots were provided as Supplementary Fig. 3. **K** Representative IHC staining showing MTHFD1 protein expression in NB patient tissue. Scale bars: 100 μm. **L** Kaplan–Meier analysis of OS or PFS for 57 NB patients with low vs high expression of MTHFD1 at our center. *P < 0.05, **P < 0.01, ***P < 0.001, ****P < 0.0001.

To identify the potential pathogenic genes associated with MYCN-amplified NB, publicly available genomic data from the TCGA database were firstly analyzed, which included 66 cases of MYCN-amplified NB and 177 cases of MYCN-non-amplified NB. The DEGs between the two groups were screened out, including 697 up-regulated genes and 1298 down-regulated genes. The heat map and volcano plot of these DEGs were shown in Fig. 1A, B, respectively. The KEGG functional enrichment analysis on the 697 significantly upregulated DEGs suggested that they were mainly enriched in folate-carbon metabolism, cell cycle, DNA replication, cysteine and methionine metabolism, glycine-serine and threonine metabolism, p53 pathway, etc. (Fig. 1C).

Folic acid metabolism is an important part of one-carbon metabolism, and is associated with high invasiveness signature of high-risk NB [17]. However, the regulatory mechanism of one-carbon metabolism in NB and its relationship with MYCN have not been clarified yet, thus the one-carbon metabolism pathway was selected for further study. Eight genes were enriched in this pathway: MTHFD1, MTHFD2, ATIC, MTHFD2L, SHMT2, DHFR, MTHFD1L and TYMS. Among them, MTHFD1 was one of the significantly upregulated DEGs. There were accumulating evidences of MTHFD1 in folate metabolism regulation of tumorigenesis, which intrigued strong research interests in recent years [18, 19]. Even though Xia Y. et al. has identified the overexpression of MTHFD1 in MYCN-amplified NB, no further validations were provided [20]. To investigate the exact role and the underlying mechanism of MTHFD1 in NB, it was selected as the object of interest for further exploration.

Based on the TCGA database, a significantly higher expression of MTHFD1 was found in the MYCN-amplified group as compared with the MYCN-non-amplified group (*P* < 0.0001, Fig. 1D). Besides, a high expression of MTHFD1 implied a worse prognosis (*P* = 0.0022, Fig. 1E). As shown by Spearman correlation analysis based on the TCGA database, MTHFD1 was positively correlated with MYCN at the transcriptional level in NB (*P* < 0.01, Fig. 1F). The results of RT-qPCR on 21 fresh tumor tissue samples of NB patients from our center further indicated a positive correlation between MTHFD1 and MYCN at the transcriptional level (*R*^2^ = 0.6690, *P* < 0.0001, Fig. 1G). In addition, 6 NB patients with MYCN amplification were verified with a high expression of MYCN protein, and a relatively higher level of MTHFD1 (Fig. 1H). Based on the above, it is speculated that MTHFD1 expression is positively correlated with MYCN in NB.

Then, the expression levels of MTHFD1 were validated in 2 MYCN-amplified NB cell lines (SK-N-BE(2) and IMR32) and 3 MYCN-non-amplified NB cell lines (SK-N-AS, SK-N-SH and SHSY-5Y). It was found by RT-qPCR and WB that the expression level of MTHFD1 in MYCN-amplified NB cells was significantly higher than that in MYCN-non-amplified NB cells, indicating a positive correlation between MTHFD1 and MYCN in NB cell lines (Fig. 1I, J).

To further verify the clinical significance and prognostic value of MTHFD1 expression level in NB, IHC staining was performed on 57 tumor samples from NB patients at our center (Fig. 1K). Then Kaplan-Meier survival analyses (Fig. 1L) revealed that NB patients exhibiting high- expression levels of MTHFD1 had worse OS and PFS than those with low-expression levels of MTHFD1. The correlation between MTHFD1 expression and the clinicopathological characteristics was analyzed among 57 NB patients (Supplementary Table 3). These results indicated that there was no significant relationship between MTHFD1 expression and patients’ age, gender, INSS stage, COG risk classification or even amplification status of MYCN. It was worth noting that, the *P* value between MTHFD1 expression and MYCN status was 0.058 in our observation, which ought to be verified with a larger sample size of tumor tissues. According to the results above, it is concluded that MTHFD1 is upregulated in MYCN-amplified NB, and a high MTHFD1 expression is associated with a poor prognosis in NB patients.

### MTHFD1 regulated the proliferation, apoptosis and migration of NB cells in vitro

**Fig. 2:**
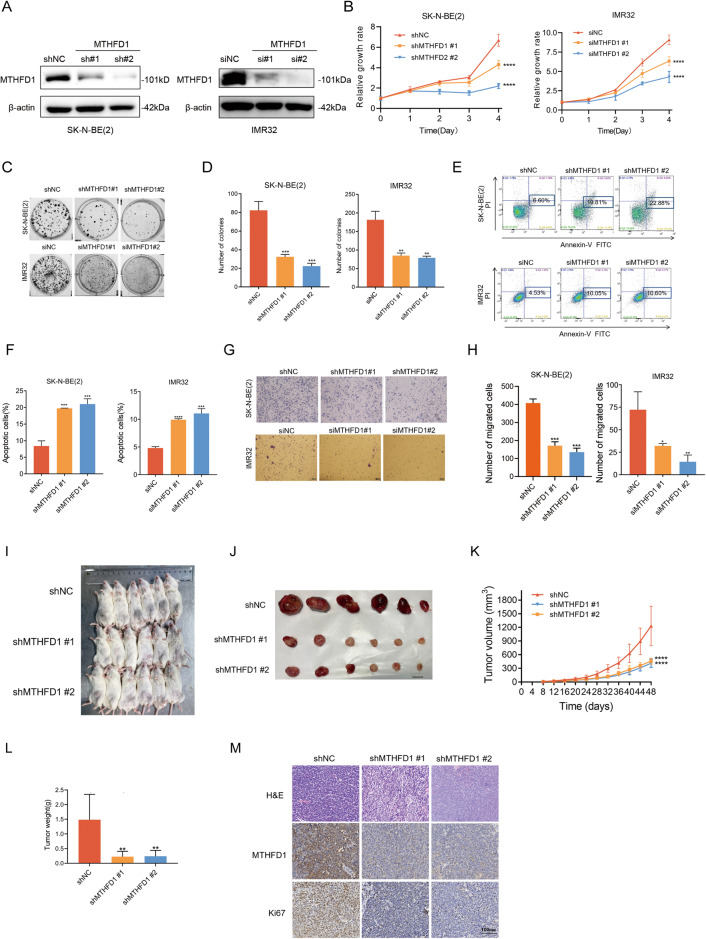
Knockdown of MTHFD1 in NB exerted anti-tumor effect both in vitro and in vivo. **A** WB analysis evaluating the knockdown efficiency of MTHFD1 with shRNAs in SK-N-BE(2) and siRNAs in IMR32 cells. The original full length western blots were provided as Supplementary Fig. 4. **B** CCK-8 assay of viability in SK-N-BE(2) and IMR32 cells transfected with MTHFD1 shRNA, MTHFD1 siRNA or the scramble control. **C** Colony formation images of MTHFD1 knockdown in SK-N-BE(2) and IMR32. **D** Histogram representative of colony formation assays in SK-N-BE(2) and IMR32 cells after MTHFD1 knockdown. **E**, **F** Changes of apoptosis rates in SK-N-BE(2) and IMR32 cells after MTHFD1 genetic knockdown. Apoptotic cells detected in three independent experiments. **G**, **H** Transwell migration assays demonstrating that knockdown of MTHFD1 decreases the migratory abilities of NB cells. **I** Xenograft tumors were established in NCG mice subcutaneously implanted with control and MTHFD1 knockdown NB cells. **J** Photograph and comparison of excised tumor size. **K** Tumor volumes were recorded on the indicated days. **L** The tumor weights were measured. **M** Representative IHC images were stained with hematoxylin and eosin (H&E) or MTHFD1 and Ki67 antibodies (Scale bars: 100 μm). *P < 0.05, **P < 0.01, ***P < 0.001, ****P < 0.0001.

To investigate the potential function of MTHFD1 in NB, 2 MYCN-amplified NB cells (SK-N-BE(2) and IMR32) were selected to first validate the effects of MTHFD1 knockdown on their biological phenotypes. The knockdown efficiency of MTHFD1 was verified with WB (Fig. 2A). The results of CCK-8 assay indicated that NB cells proliferated at a significantly slower rate after genetic knockdown of MTHFD1 (Fig. 2B). Colony formation assay also revealed the anti-proliferation effects on SK-N-BE(2) and IMR32 cells after MTHFD1 suppression (Fig. 2C, D). The apoptosis of SK-N-BE(2) and IMR32 cells was significantly induced (Fig. 2E, F), while the migration ability of NB cells was obviously weakened after knockdown of MTHFD1 (Fig. 2G, H).

Besides, the regulation of MTHFD1 in NB biological behaviors was further verified in the MYCN-non amplified NB cell SK-N-AS. It was found that the ectopic overexpression of MTHFD1 (Supplementary Fig. 1A) obviously enhanced the proliferation (Supplementary Fig. 1B, C) and migration (Supplementary Fig. 1D), but reduced the apoptosis (Supplementary Fig. 1E) of NB cells.

To sum up, MTHFD1 exerts as an oncogene and promotes the tumorigenesis of NB in vitro.

### MTHFD1 exerted a tumorigenic effect in vivo

To further determine the tumorigenic effect of MTHFD1 in vivo, the MYCN-amplified NB SK-N-BE(2) cells were subcutaneously injected into the NCG mice to establish the xenograft model. The mice were divided into 3 groups: shNC, shMTHFD1#1 and shMTHFD1#2 (Fig. 2I, J). As shown in Fig. 2K, the tumors in shNC group grew faster than those in the other two groups. Moreover, tumor weight in shNC group was larger than that in the other two groups (Fig. 2L), suggesting an anti-proliferation effect of MTHFD1 knockdown. IHC staining validated that the tumors had lower expressions of Ki67 after MTHFD1 knockdown (Fig. 2M), which indicated a lower proliferation rate.

### MTHFD1 was transcriptionally activated by MYCN in NB

**Fig. 3:**
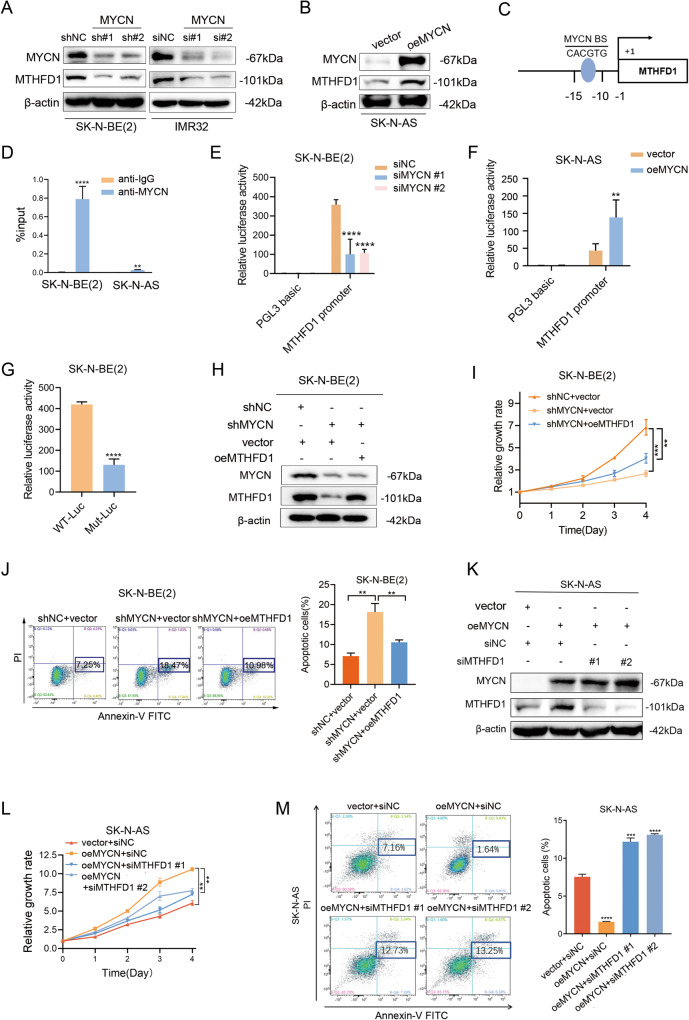
MTHFD1 was transcriptional activated by MYCN in NB. **A** WB analysis of MTHFD1 protein expression in SK-N-BE(2) and IMR32 cells with MYCN knockdown. The original full length western blots were provided as Supplementary Fig. 5. **B** WB analysis of MTHFD1 protein expression in SK-N-AS cells with MYCN overexpression. The original full length western blots were provided as Supplementary Fig. 5. **C** Schematic diagram showing the location of potential MYCN binding sites on the MTHFD1 promoter. **D** ChIP-qPCR indicated the enrichment of MYCN at MTHFD1 promoter region in SK-N-BE(2) and SK-N-AS cells. **E** Dual-luciferase reporter assay showed the luciferase activity of MTHFD1 after genetic knockdown of MYCN in SK-N-BE(2). **F** Relative MTHFD1 luciferase promoter activity in SK-N-AS cells with MYCN overexpression. **G** Relative luciferase activity of constructs including the MTHFD1 promoter (WT-Luc) or the mutation of MYCN binding site (Mut-Luc) in SK-N-BE(2) cells. **H** WB analysis of MYCN expression in MYCN-knockdown SK-N-BE(2) cells after MTHFD1 overexpression. The original full length western blots were provided as Supplementary Fig. 6. **I** CCK-8 assay of viability in MYCN-knockdown SK-N-BE(2) cells with MTHFD1 overexpression. **J** Changes of apoptosis rates in MYCN-knockdown SK-N-BE(2) cells after MTHFD1 overexpression. **K** WB analysis of MTHFD1 expression in MYCN-overexpressed SK-N-AS cells after MTHFD1 knockdown. The original full length western blots were provided as Supplementary Fig. 6. **L** CCK-8 assay of viability in MYCN-overexpressed SK-N-AS cells with MTHFD1 knockdown. **M** Changes of apoptosis rates in MYCN-overexpressed SK-N-AS cells after MTHFD1 genetic knockdown. **P < 0.01, ***P < 0.001, ****P < 0.0001.

The above findings indicated the tumorigenic effect of MTHFD1 on MYCN-amplified NB cells both in vitro and in vivo. Meanwhile, MTHFD1 was positively correlated with MYCN at the mRNA and protein levels. As previously reported, the expression of MTHFD1 is significantly high in MYCN-amplified NB, but no further verification has been performed [20]. As a member of the MYC transcription factor family, MYCN is dysregulated in various tumors and regulates the transcription of multiple oncogenes [21, 22]. Based on these findings, it is speculated that MTHFD1 may be the target gene of MYCN. To verify our speculation, MYCN was firstly knocked down in MYCN-amplified NB cells. The down-regulation of MTHFD1 was found, consistent with the results of WB (Fig. 3A). Then MYCN was overexpressed in MYCN-non-amplified NB SK-N-AS cell lines, and the up-regulation of MTHFD1 was observed as expected (Fig. 3B). These results revealed that MYCN regulates the MTHFD1 expression in NB.

To further investigate the possible molecular mechanisms by which MYCN regulates MTHFD1 expression, the possible binding sites to MYCN at the MTHFD1 promoter regions (about 1500 bp upstream from the transcription start site) were predicted based on the PROMO online database (http://alggen.lsi.upc.es/cgibin/promo_v3/promo/promoinit.cgi?dirDB=TF_8.3/), and a potential binding site to MYCN was found (5’-CACGTG-3’) (Fig. 3C). Then ChIP-qPCR results indicated that MYCN was enriched at the MTHFD1 promoter region. Compared with the MYCN-non-amplified SK-N-AS cells, the MYCN-amplified SK-N-BE(2) cells showed an enhanced enrichment at the promoter region of MTHFD1 (Fig. 3D). The results of dual-luciferase reporter assay demonstrated that the activation of MTHFD1 promoter was significantly decreased after knockdown of MYCN (Fig. 3E), while an obviously enhanced promoter activity of MTHFD1 was observed after overexpression of MYCN in SK-N-AS cells (Fig. 3F). When the MTHFD1 promoter binding site (5’-CACGTG-3’) was mutated to 5’-ACATGT-3’, the promoter activity was significantly reduced in mutant group compared with that in wild-type group (Fig. 3G). Rescue assays showed that: after MYCN knockdown, the re-introduced MTHFD1(Fig. 3H) could partially reverse the reduced proliferation abilities (Fig. 3I) and the increased apoptosis (Fig. 3J) of SK-N-BE(2). Accordingly, knockdown of MTHFD1 with siRNA partially reversed (Fig. 3K) the proliferation-promoting effect (Fig. 3L) and the anti-apoptosis effect (Fig. 3M) of SK-N-AS resulting from the overexpression of MYCN. The results above demonstrated that MTHFD1 is a direct target gene of MYCN and can be transcriptionally upregulated by MYCN.

### MTHFD1 maintained redox homeostasis in NB

**Fig. 4:**
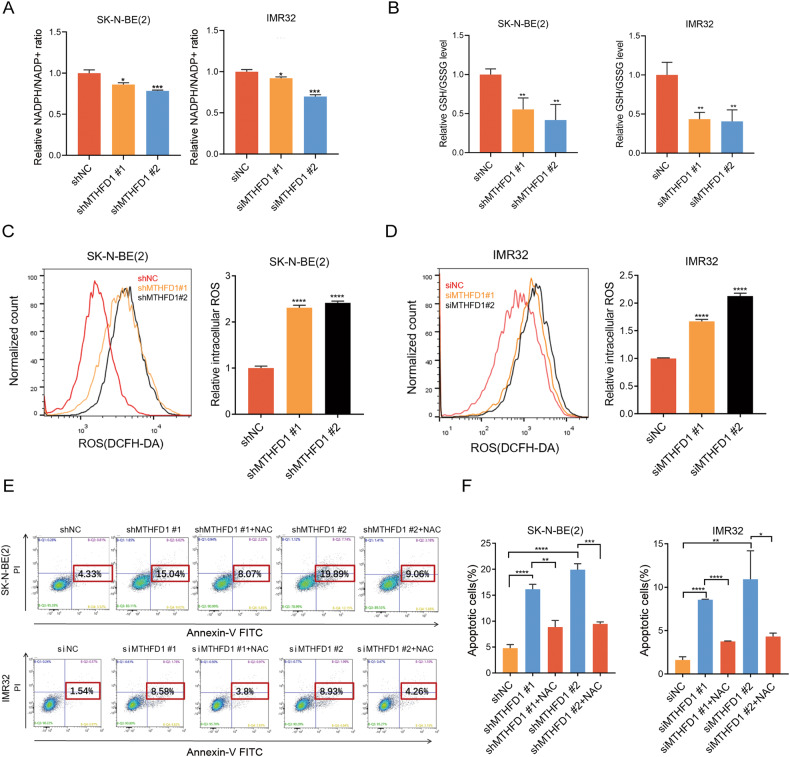
MTHFD1 increased NADPH/NADP+ levels and GSH/GSSG levels, reduced ROS levels. **A** Levels of NADPH/NADP+ in SK-N-BE(2) and IMR32 cells with or without MTHFD1 knockdown. **B** Levels of GSH/GSSG in SK-N-BE(2) and IMR32 cells with or without MTHFD1 knockdown. **C** Levels of intracellular ROS in SK-N-BE(2) cells with or without MTHFD1 knockdown. **D** Levels of intracellular ROS in IMR32 cells with or without MTHFD1 knockdown. **E**, **F** Changes of apoptosis rates in SK-N-BE(2) and IMR32 cells after MTHFD1 genetic knockdown in the presence or absence of NAC. *P < 0.05, **P < 0.01, ***P < 0.001, ****P < 0.0001.

MTHFD1 has been found to induce NADPH production and reduce the ROS content in cholangiocarcinoma cells [23]. MTHFD1 can also promote tumorigenesis and lead to drug resistance by regulating NADPH homeostasis in the one-carbon metabolism process in acute myeloid leukemia [24]. The influence of MTHFD1 on the folic acid metabolism in NB has not been elucidated yet. Here we investigated the role of MTHFD1 in regulating NADPH redox homeostasis in NB. It was found that suppression of MTHFD1 reduced both NADPH/NADP^+^ and GSH/GSSG ratios (Fig. 4A, B), and obviously increased cellular ROS content in SK-N-BE(2) and IMR32 cells (Fig. 4C, D).

Excessive intracellular ROS can induce cytotoxicity and lead to apoptosis of tumor cells [25, 26]. The results revealed that MTHFD1 knockdown induced apoptosis of NB cells (Fig. 2E, F). To investigate whether MTHFD1 affects apoptosis through redox homeostasis in NB, the antioxidant N-Acetyl-L-cysteine (NAC) was used after MTHFD1 knockdown in NB cells. It was found that the enhanced apoptosis caused by MTHFD1 knockdown could be partially reversed by NAC in NB cells (Fig. 4E, F), indicating that MTHFD1 affects apoptosis through regulating redox homeostasis.

### Knockdown of MTHFD1 enhanced the anti-tumor effect of JQ1 in NB

As an inhibitor of the folic acid metabolism pathway, methotrexate (MTX) is a widely used anti-cancer agent in the treatment of multiple childhood and adult cancers. Diana TL et al. has demonstrated that MYCN-amplified NB cells have a higher requirement for folate than MYCN-non-amplified NB cells, the MYCN-amplified NB cells like SK-N-BE(2) and IMR32 are more sensitive to MTX than other NB cell lines without MYCN amplification [27]. Another study by Ken Y et al. also verified the higher sensitivity of MYCN-amplified NB cells after MTX treatment [28]. Besides, MTX can lead to a decrease in the expression level of MTHFD1 [19].

As the BRD4 inhibitor, JQ1 can reduce the expression of MYCN and inhibit tumor growth in MYCN-amplified NB, but its effect seems unsatisfactory when used alone [3]. Notably, the combination of MTX and JQ1 exerts a significantly synergistic anti-tumor effect in hematologic tumors [19]. Based on the above, the effect of inhibiting the folic acid metabolism by silencing MTHFD1 or MTX treatment, combinating with suppressing MYCN expression by JQ1 was then investigated.

**Fig. 5:**
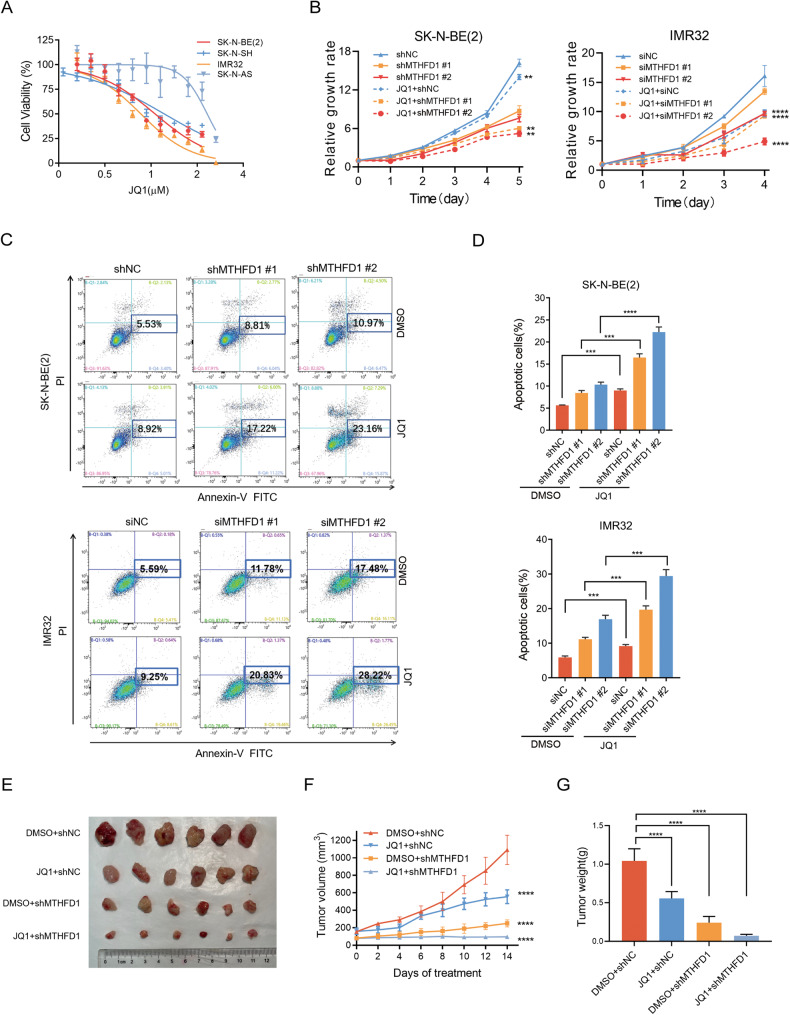
Knockdown of MTHFD1 enhanced the anti-tumor effect of JQ1 in NB. **A** The cell viability of the indicated NB cells treated with JQ1 for 72 h was analyzed using CCK-8 assay. **B** Proliferation of NB cells with or without MTHFD1 knockdown in the presence of vehicle control (Ctrl) and 0.2 μM JQ1. **C**, **D** Changes of apoptosis rates in SK-N-BE(2) and IMR32 cells with or without MTHFD1 knockdown treated with vehicle control or JQ1 for 48 h. **E** Pictures of subcutaneous xenografts. **F** Growth curves of subcutaneous xenografts derived from SK-N-BE(2) shNC and shMTHFD1 cells in NCG mice that were administered vehicle control (Ctrl) or 50 mg/kg/d JQ1. **G** Quantification of tumor mass. **P < 0.01, ***P < 0.001, ****P < 0.0001.

Results of the CCK-8 assay showed that the MYCN-amplified NB cells (SK-N-BE(2) and IMR32) had lower IC50 values, suggesting that they are more sensitive to JQ1 (Fig. 5A). JQ1 at a concentration of 0.1 μM was selected for further experiments. The growth curves revealed that JQ1 significantly enhanced the growth inhibition induced by MTHFD1 knockdown in a time-dependent manner (Fig. 5B). JQ1 could also distinctly enhance the apoptosis induced by MTHFD1 suppression in SK-N-BE(2) and IMR32 cells (Fig. 5C, D). Furthermore, mouse model experiments validated that the inhibitory effect of MTHFD1 knockdown on proliferation was significantly strengthened in NB cells treated with JQ1 (Fig. 5E–G).

### MTX augmented the anti-tumor effect of JQ1 in NB

**Fig. 6:**
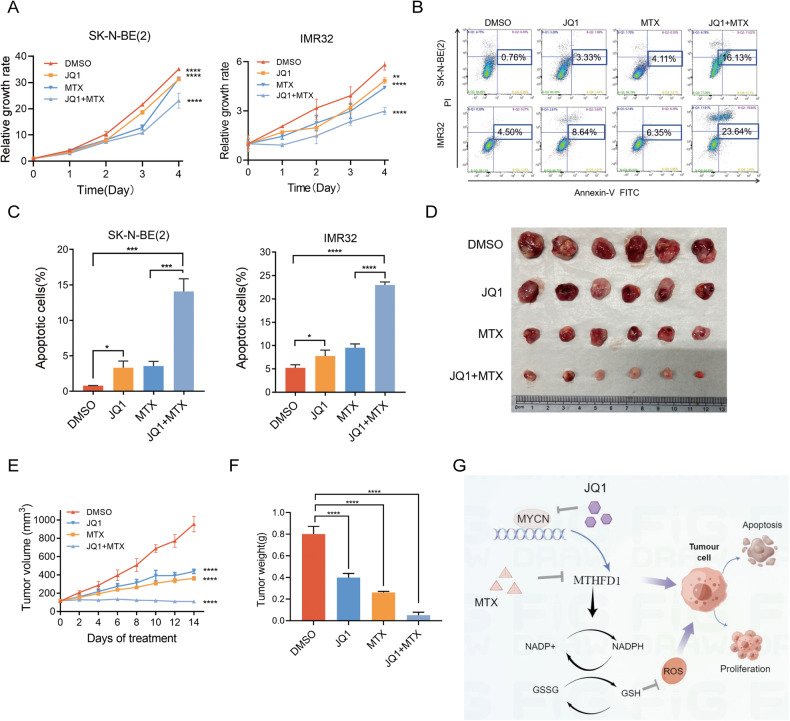
MTX augmented the anti-tumor effect of JQ1 in NB. **A** Proliferation of SK-N-BE(2) cells treated with vehicle control (Ctrl), 0.2 μM JQ1, 0.2 μM MTX, 0.2 μM JQ1 + 0.2 μM MTX. **B** Proliferation of IMR32 cells treated with vehicle control (Ctrl), 0.2 μM JQ1, 0.2 μM MTX, 0.2 μM JQ1 + 0.2 μM MTX. **C** Changes of apoptosis rates in SK-N-BE(2) and IMR32 cells treated with vehicle control, 0.2 μM MTX or 0.2 μM JQ1 for 72 h. **D** Pictures of subcutaneous xenografts. **E** Growth curves of subcutaneous xenografts administered with vehicle control (Ctrl), 50 mg/kg/d JQ1 and/or twice weekly with MTX 20 mg/kg/d. **F** Quantification of tumor mass. **G** The graphic illustration of regulatory mechanism diagram of our study. *P < 0.05, **P < 0.01, ***P < 0.001, ****P < 0.0001.

Next, the combination effect of JQ1 and the folic acid inhibitor MTX was explored. MTX at a working concentration of 0.15 μM was used. The results of experiments in vitro demonstrated that in JQ1 + MTX group, the inhibitory effect on cell proliferation was more obvious than that in single drug group (Fig. 6A), and the proportion of apoptotic SK-N-BE(2) and IMR32 cells was dramatically increased (Fig. 6B, C). Consistently, experiments in vivo also validated that the combination of JQ1 and MTX caused significant proliferation suppression of MYCN-amplified NB (Fig. 6D–F).

To sum up, MTHFD1 is an oncogene in MYCN-amplified NB, and it regulates redox homeostasis and promotes the malignant progression of NB. In terms of the mechanism, MTHFD1 is transcriptionally upregulated by MYCN. Inhibition of MTHFD1 augments the anti-tumor effect of JQ1 in MYCN-amplified NB (Fig. 6G).

## Discussion

MYCN amplification is an important risk stratification factor for NB. Inhibition of MYCN seems to be a promising therapeutic approach to improve the outcome of patients with high-risk NB. However, due to the special α-helical structure on the surface of MYCN protein, developing small molecule inhibitors directly targeting MYCN remains challenging. At present, researchers are enthusiastically searching for key transcriptional targets at the downstream of MYCN, aiming to explore treatment options for MYCN-amplified NB. In this study, the DEGs between MYCN-amplified and MYCN-non-amplified NB patients were identified by bioinformatics analysis based on the TCGA database. The KEGG functional enrichment analysis showed that carbon metabolism was one of the main pathways with significant changes, among which MTHFD1 was the major DEG. Subsequently, bioinformatics analysis and verification in both NB tissues and cell lines confirmed the positive correlation between MTHFD1 and MYCN. MTHFD1 was highly expressed in MYCN-amplified NB, and a high expression of MTHFD1 was associated with a poor prognosis of NB patients, suggesting that MTHFD1 may be associated with the malignant progression of MYCN-amplified NB.

Previous studies indicated that MTHFD1 is highly expressed in a variety of tumors and plays crucial roles in the malignant progression of tumors [24, 29]. In cholangiocarcinoma, MTHFD1 reduces the ROS content in tumor cells, and induces resistance to gemcitabine, thus enhancing the progressive phenotype of tumors [23]. MTHFD1 maintains redox homeostasis in the folic acid metabolism process in metastatic colorectal cancer cells and serves as a potential therapeutic target for colorectal cancer [30]. In lung cancer, knockdown of MTHFD1 significantly increases the percentage of apoptotic tumor cells [31]. In this study, it was found that MTHFD1 played a tumor-promoting role in MYCN-amplified NB, and it promoted proliferation and migration but inhibited apoptosis of NB cells. Animal experiments in mice also revealed the tumorigenic role of MTHFD1 in MYCN-amplified NB cells in vivo. These results were consistent with previous findings that MTHFD1 serves as an oncogene in tumors.

Recent studies have reported that metabolic reprogramming in MYCN-amplified NB is dependent on the activation of one-carbon metabolism pathway, and MTHFD1 is identified as one of the significant DEGs in this pathway based on the sequencing results [20, 32, 33]. In addition, several key enzymes in the carbon metabolism process are highly expressed in NB and exert a tumor-promoting effect [34–37]. These findings suggested that the carbon metabolism pathway is abnormally activated in MYCN-amplified NB. In this study, it was also verified that the carbon metabolism pathway was activated in MYCN-amplified NB. Subsequent experiments confirmed that MYCN could bind to the MTHFD1 promoter region and transcriptionally activate the expression of MTHFD1 in MYCN-amplified NB, validating the molecular mechanism of MYCN regulating MTHFD1. Targeted inhibition of MTHFD1 may provide a promising therapy for MYCN-amplified NB.

Folic acid metabolism belongs to carbon metabolism, during which several enzymes are considered potential tumor-specific therapeutic targets. The folic acid metabolizing enzyme MTHFD1L can generate NADPH to defend against oxidative stress and promote tumor growth in liver cancer [38]. ATF4 and c-MYC promote tumor cell growth by synergistically regulating MTHFD2, and MTHFD2 is identified as a biomarker or therapeutic target for prostate cancer [39]. Inhibiting MTHFD1 in chronic myelogenous leukemia causes a significant decrease in the proliferative ability of tumor cells both in vitro and in vivo [19]. Similarly, knocking down MTHFD1 can reduce the antioxidant stress ability of tumor cells, thus inhibiting the distant metastasis of melanoma [40].

As a key enzyme in folic acid metabolism, MTHFD1 plays an important role in maintaining redox homeostasis, NADPH production and nucleotide metabolism. NADPH is important for ROS generation and catalyzes the GSSG reaction, thereby producing GSH and then inducing ROS generation. A high level of ROS in cells causes DNA damage and mitochondrial activation, inducing cell senescence and apoptosis [41, 42]. However, the regulatory effect of MTHFD1 on redox homeostasis in NB has not been elucidated yet. In this study, it was verified that knockdown of MTHFD1 decreased both NADPH/NADP^+^ and GSH/GSSG ratios and increased cellular ROS content in SK-N-BE(2) and IMR32 cells. The results suggested that MTHFD1 maintained redox homeostasis in MYCN-amplified NB, and inhibiting MTHFD1 in tumor cells may weaken the resistance to oxidative stress, thus preventing the malignant progression of tumors.

MTX is the inhibitor of folic acid metabolism, which can cause the loss of chromatin-associated MTHFD1 [19, 23, 24], but the anti-tumor effect of MTX alone in NB seems unsatisfactory due to high toxicity and side effects [43, 44]. Despite this, its anti-tumor effect in MYCN-amplified NB is better than that in MYCN-non-amplified NB. The BET inhibitor JQ1 can down-regulate the MYCN expression in MYCN-amplified NB [3], but its effect when used alone is also poor. Interestingly, when combining used MTX and JQ1 in MYCN-amplified NB, a synergistic anti-tumor effect was found in our study. When the folic acid metabolism was inhibited by MTHFD1 knockdown, an anti-NB effect of JQ1 was enhanced as well. Our results were in accord with the reported study that combination of MTX and JQ1 could synergistically inhibit the proliferation of CML cells both in vitro and in vivo [19], which provided a preliminary basis for the treatment of MYCN-amplified NB with the combination of these two drugs.

In conclusion, this study revealed the tumor-promoting role of MTHFD1 in NB, and elucidated the molecular mechanism by which MTHFD1 was directly transcriptionally regulated by MYCN. MTHFD1 maintains redox homeostasis and protects tumor cells from oxidative stress. Genetic knockdown of MTHFD1 or application of folic acid inhibitor MTX combined with JQ1 can synergistically inhibit tumor progression in MYCN-amplified NB. Thus, MTHFD1 may be a potential therapy target for MYCN-amplified NB.

### Supplementary information

Supplementary Fig 1–7

Table S1

Table S2–3

Original Data File

Original Data File

checklist

## Data Availability

All the data of the findings from the present study have been validated by uploading crucial raw data to the public platform (www.researchdata.org.cn) of Research Data Deposit (RDD), with the number of RDDB2023339777, and are available from the corresponding author under reasonable request.
