# Supplementary material for: MTHFD1 regulates the NADPH redox homeostasis in MYCN-amplified neuroblastoma
Source: Cell Death Dis. 2024 Feb 9;15(2):124. doi: 10.1038/s41419-024-06490-3 (PMC10858228; doi:10.1038/s41419-024-06490-3)
Supplement: Supplementary file 1 — Supplementary Fig 1–7 [file 41419_2024_6490_MOESM1_ESM.pdf]

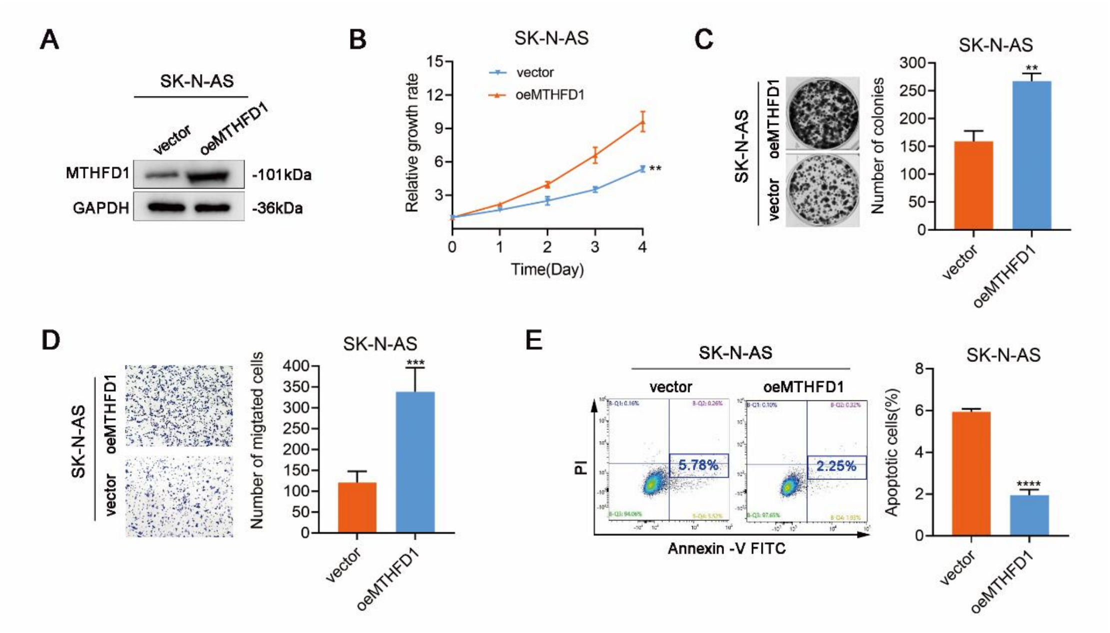

Supplementary Fig 1. The tumorigenesis effect of MTHFD1 in SK-N-AS cells with MTHFD1 overexpression.

(A) WB analysis verified the transfection efficiency in SK-N-AS cells with MTHFD1 overexpression. The original full length western blots were provided as Supplementary Fig 7.

(B) The proliferation analysis of SK-N-AS cell with MTHFD1 overexpression.

(C) The colony formation assay of SK-N-AS cell with MTHFD1 overexpression.

(D) Transwell migration assay in SK-N-AS cell with MTHFD1 overexpression.

(E) Changes of apoptosis rates in SK-N-AS cell with MTHFD1 overexpression.

**$P < 0.01$, ***$P < 0.001$, ****$P < 0.0001$.

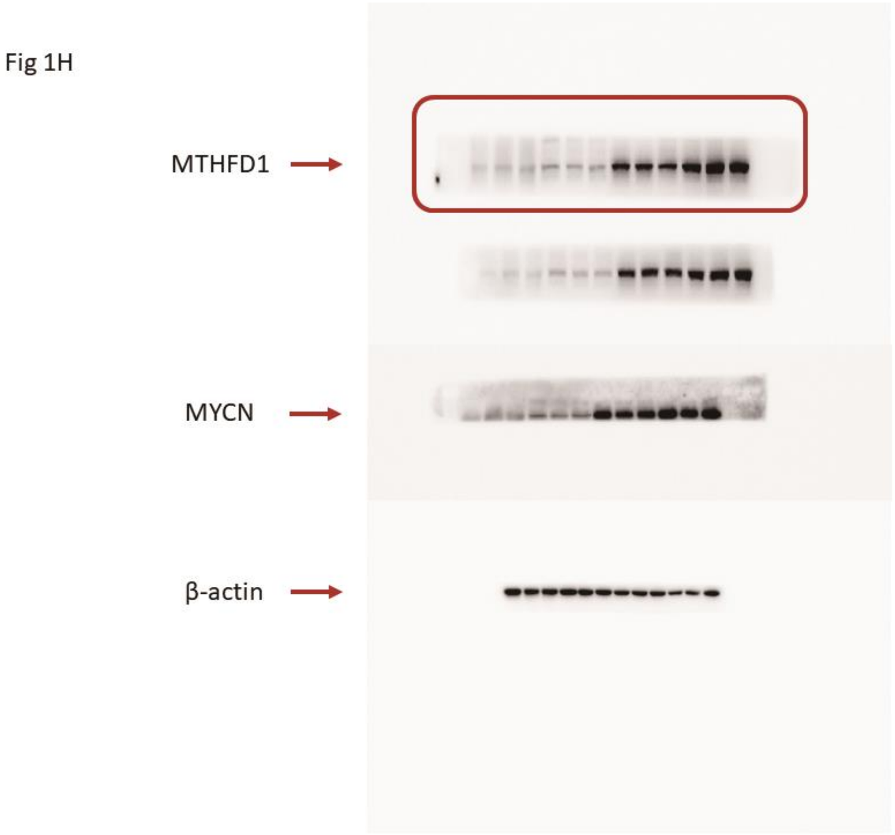

Supplementary Fig 2. The original full length western blots of Fig 1H.

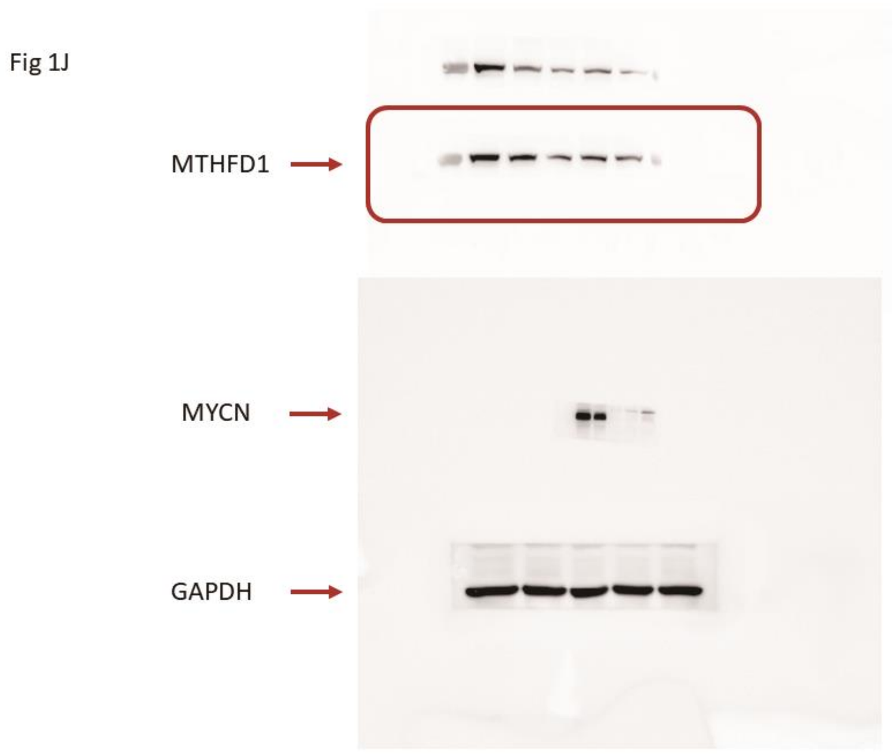

Supplementary Fig 3. The original full length western blots of Fig 1J.

Fig 2A

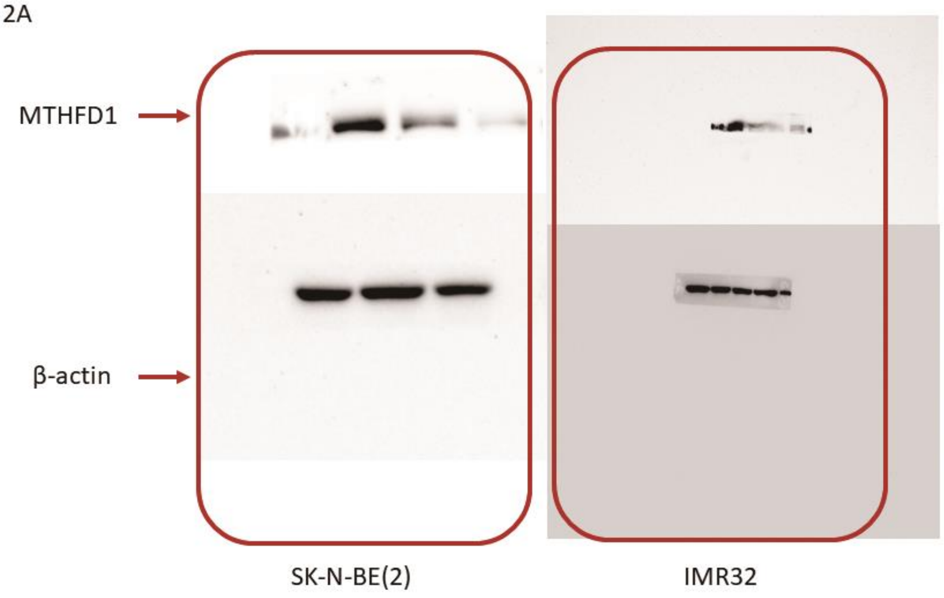

Supplementary Fig 4. The original full length western blots of Fig 2A.

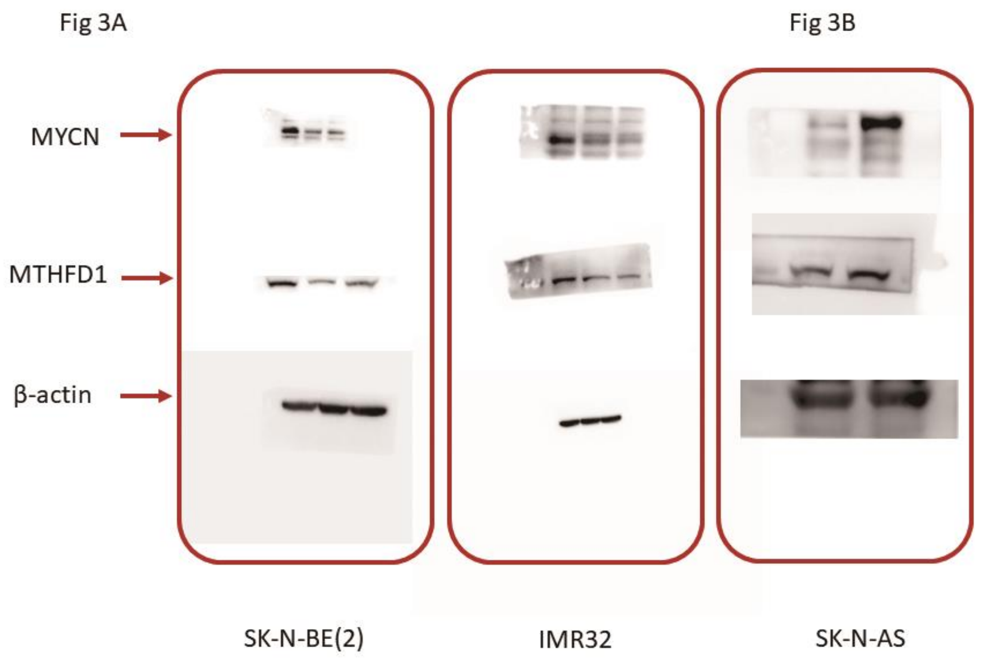

Supplementary Fig 5. The original full length western blots of Fig 3A, 3B.

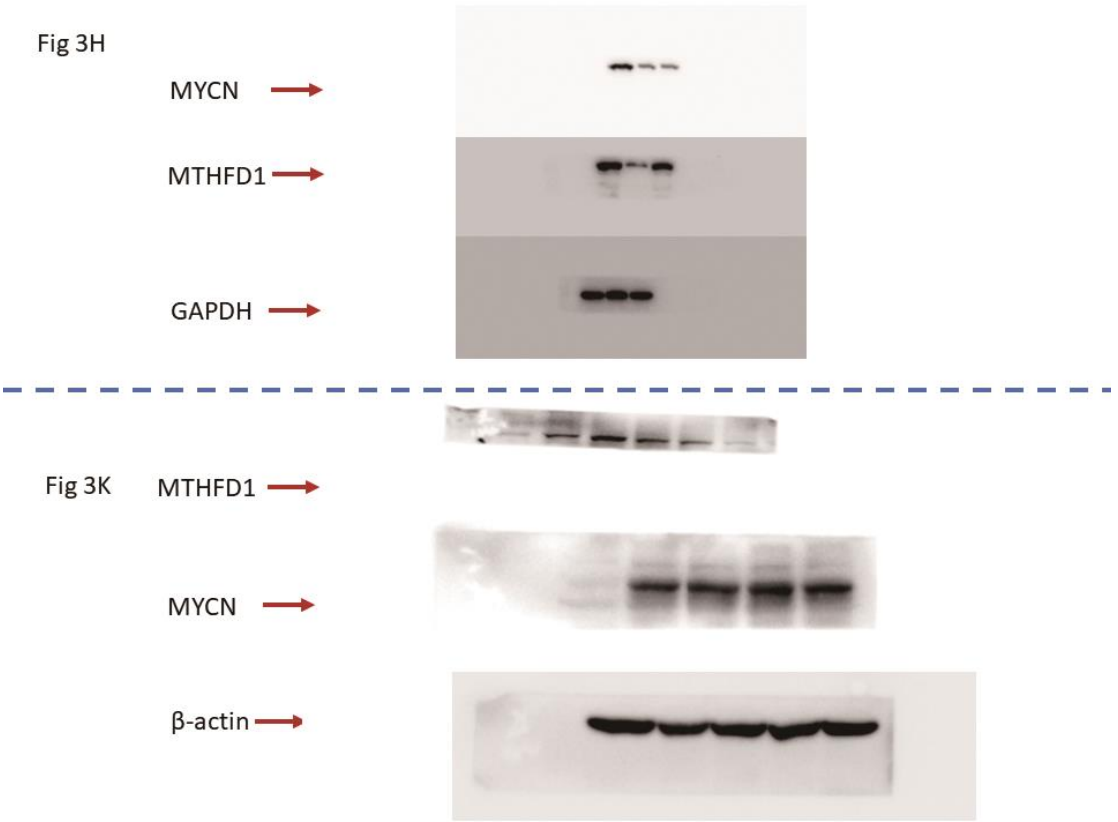

Supplementary Fig 6. The original full length western blots of Fig 3H，3K.

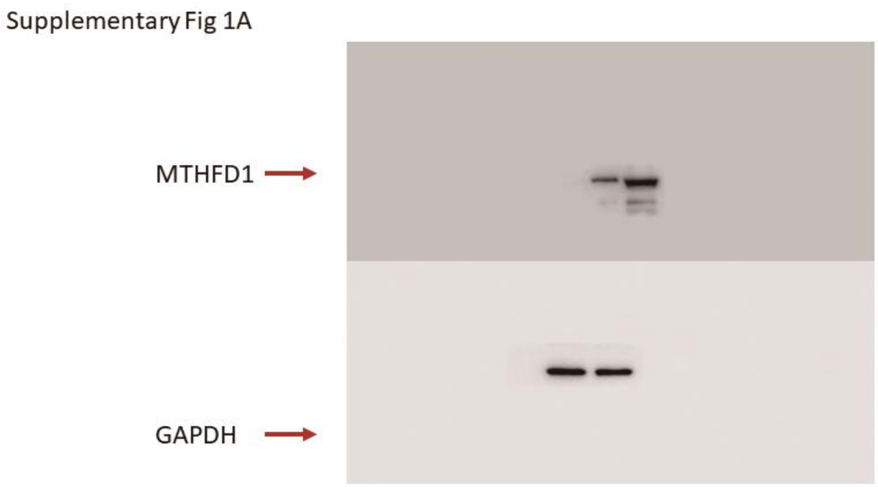

Supplementary Fig 7. The original full length western blots of Supplementary Fig 1A.
