# Supplementary material for: MTHFD1 regulates the NADPH redox homeostasis in MYCN-amplified neuroblastoma
Source: Cell Death Dis. 2024 Feb 9;15(2):124. doi: 10.1038/s41419-024-06490-3 (PMC10858228; doi:10.1038/s41419-024-06490-3)
Supplement: Supplementary file 2 — Table S1 [file 41419_2024_6490_MOESM2_ESM.docx]

**Supplementary Table 1.** **Clinical characteristics of patients with neuroblastoma.**

| **Patient number** | **MYCN amplification** | | **MTHFD1 expression** | **Gender** | **Age (years old)** | | **INPC** | **INSS stage** | **COG risk classification** |
| --- | --- | --- | --- | --- | --- | --- | --- | --- | --- |
| T1 | | Yes | Negative | Male | > 1.5 | Unfavorable histology | | 4 | High risk |
| T2 | | Yes | Negative | Male | ≤ 1.5 | Unfavorable histology | | 4 | High risk |
| T3 | | Yes | Negative | Male | > 1.5 | Unfavorable histology | | 4 | High risk |
| T4 | | Yes | Negative | Male | > 1.5 | Unfavorable histology | | 4 | High risk |
| T5 | | Yes | Negative | Female | > 1.5 | Unfavorable histology | | 3 | High risk |
| T6 | | Yes | Negative | Male | > 1.5 | Unfavorable histology | | 4 | High risk |
| T7 | | Yes | Positive | Female | > 1.5 | Unfavorable histology | | 4 | High risk |
| T8 | | Yes | Positive | Male | > 1.5 | Unfavorable histology | | 4 | High risk |
| T9 | | Yes | Positive | Male | > 1.5 | Unfavorable histology | | 4 | High risk |
| T10 | | Yes | Positive | Female | > 1.5 | Favorable histology | | 3 | High risk |
| T11 | | Yes | Positive | Female | > 1.5 | Unfavorable histology | | 1 | Low risk |
| T12 | | Yes | Positive | Female | > 1.5 | Unfavorable histology | | 4 | High risk |
| T13 | | Yes | Negative | Female | > 1.5 | Unfavorable histology | | 2 | High risk |
| T14 | | Yes | Negative | Female | > 1.5 | Unfavorable histology | | 4 | High risk |
| T15 | | Yes | Negative | Female | > 1.5 | Unfavorable histology | | 4 | High risk |
| T16 | | Yes | Negative | Male | > 1.5 | Unfavorable histology | | 4 | High risk |
| T17 | | Yes | Negative | Male | > 1.5 | Unfavorable histology | | 4 | High risk |
| T18 | | No | Negative | Male | > 1.5 | Unfavorable histology | | 4 | High risk |
| T19 | | No | Negative | Female | ≤ 1.5 | Unfavorable histology | | 4 | High risk |
| T20 | | No | Negative | Male | > 1.5 | Unfavorable histology | | 2 | Low risk |
| T21 | | No | Negative | Female | > 1.5 | Favorable histology | | 2 | Low risk |
| T22 | | No | Negative | Male | > 1.5 | Unfavorable histology | | 1 | Low risk |
| T23 | | No | Negative | Male | ≤ 1.5 | Unfavorable histology | | 4 | High risk |
| T24 | | No | Negative | Female | > 1.5 | Unfavorable histology | | 4 | High risk |
| T25 | | No | Negative | Male | ≤ 1.5 | Unfavorable histology | | 4 | Intermediate risk |
| T26 | | No | Negative | Female | > 1.5 | Favorable histology | | 4 | High risk |
| T27 | | No | Negative | Male | > 1.5 | Favorable histology | | 4 | High risk |
| T28 | | No | Negative | Female | > 1.5 | Unfavorable histology | | 4 | High risk |
| T29 | | No | Negative | Female | > 1.5 | Unfavorable histology | | 4 | High risk |
| T30 | | No | Negative | Male | > 1.5 | Favorable histology | | 3 | Intermediate risk |
| T31 | | No | Negative | Male | > 1.5 | Unfavorable histology | | 3 | High risk |
| T32 | | No | Negative | Male | > 1.5 | Unfavorable histology | | 3 | High risk |
| T33 | | No | Negative | Female | > 1.5 | Unfavorable histology | | 4 | High risk |
| T34 | | No | Negative | Female | ≤ 1.5 | Favorable histology | | 2 | Low risk |
| T35 | | No | Negative | Female | > 1.5 | Unfavorable histology | | 2 | Low risk |
| T36 | | No | Negative | Female | ≤ 1.5 | Unfavorable histology | | 1 | Low risk |
| T37 | | No | Negative | Male | > 1.5 | Favorable histology | | 1 | Low risk |
| T38 | | No | Negative | Male | > 1.5 | Favorable histology | | 1 | Low risk |
| T39 | | No | Negative | Female | > 1.5 | Favorable histology | | 1 | Low risk |
| T40 | | No | Negative | Male | > 1.5 | Unknown | | 4 | High risk |
| T41 | | No | Negative | Male | > 1.5 | Unfavorable histology | | 4 | High risk |
| T42 | | No | Negative | Female | ≤ 1.5 | Unfavorable histology | | 4 | Intermediate risk |
| T43 | | No | Negative | Female | ≤ 1.5 | Unfavorable histology | | 4 | Intermediate risk |
| T44 | | No | Negative | Male | ≤ 1.5 | Unfavorable histology | | 4 | Intermediate risk |
| T45 | | No | Negative | Female | > 1.5 | Favorable histology | | 4 | High risk |
| T46 | | No | Negative | Male | > 1.5 | Favorable histology | | 4 | High risk |
| T47 | | No | Negative | Male | > 1.5 | Unfavorable histology | | 4 | High risk |
| T48 | | No | Negative | Male | > 1.5 | Favorable histology | | 4 | High risk |
| T49 | | No | Negative | Male | > 1.5 | Favorable histology | | 4 | High risk |
| T50 | | No | Negative | Male | > 1.5 | Unfavorable histology | | 4 | High risk |
| T51 | | No | Negative | Female | > 1.5 | Unfavorable histology | | 4 | High risk |
| T52 | | No | Negative | Male | > 1.5 | Unfavorable histology | | 4 | High risk |
| T53 | | No | Negative | Male | > 1.5 | Unfavorable histology | | 4 | High risk |
| T54 | | No | Negative | Male | > 1.5 | Unfavorable histology | | 4 | High risk |
| T55 | | No | Negative | Female | ≤ 1.5 | Favorable histology | | 3 | Intermediate risk |
| T56 | | No | Negative | Female | > 1.5 | Favorable histology | | 1 | Low risk |
| T57 | | No | Negative | Female | > 1.5 | Favorable histology | | 1 | Low risk |
