# Supplementary material for: MTHFD1 regulates the NADPH redox homeostasis in MYCN-amplified neuroblastoma
Source: Cell Death Dis. 2024 Feb 9;15(2):124. doi: 10.1038/s41419-024-06490-3 (PMC10858228; doi:10.1038/s41419-024-06490-3)
Supplement: Supplementary file 3 — Table S2–3 [file 41419_2024_6490_MOESM3_ESM.docx]

**Supplementary Table 2. Primer sequences of RT-qPCR.**

| **Primer** | **Sequence** |
| --- | --- |
| GAPDH Forward | CCTTCCGTGTCCCCACT |
| GAPDH Reverse | GCCTGCTTCACCACCTTC |
| Actin Forward | TCTCCCAAGTCCACACAGG |
| Actin Reverse | GGCACGAAGGCTCATCA |
| MTHFD1 Forward | AAAGAGAGGGCGAGCTTCAT |
| MTHFD1 Reverse | CCTCTTCAGACAGCAGACCA |
| MYCN Forward | GGGGACTGTTTCTGCTTCC |
| MYCN Reverse | AGGATTAGGGCGGGTCTC |

**Supplementary Table 3. Correlation between MTHFD1 expression and clinicopathological characteristics in 57 NB patients.**

| Variables | MTHFD1 | | *P*-value* |
| --- | --- | --- | --- |
|  | Negative expression, n=51 | Positive expression, n=6 |  |
| Gender |  |  |  |
| Male | 28（54.9） | 3（50%） | 0.576 |
| Female | 23（45.1） | 3（50%） |  |
| Age |  |  |  |
| ≤ 1.5 | 9（17.6） | 1（16.7） | 0.719 |
| > 1.5 | 42（82.4） | 5（83.3） |  |
| INSS |  |  |  |
| 1 | 8（15.7） | 0（0） | 0.562 |
| 2 | 5（9.8） | 0（0） |  |
| 3 | 5（9.8） | 1（16.7） |  |
| 4 | 33（64.7） | 5（83.3） |  |
| COG risk classification |  |  |  |
| Low risk | 12（23.5） | 0（0） | 0.213 |
| Intermediate risk | 6（11.8） | 0（0） |  |
| High risk | 33（64.7） | 6（100） |  |
| MYCN amplification |  |  |  |
| Y | 13（25.5） | 4（66.7） | 0.058 |
| N | 38（74.5） | 2（33.3） |  |

Values are presented as number（%）;

Abbreviations: Y, Yes. N, No. *Chi-square test.
