# Supplementary material for: MTHFD1 regulates the NADPH redox homeostasis in MYCN-amplified neuroblastoma
Source: Cell Death Dis. 2024 Feb 9;15(2):124. doi: 10.1038/s41419-024-06490-3 (PMC10858228; doi:10.1038/s41419-024-06490-3)
Supplement: Supplementary file 6 — checklist [file 41419_2024_6490_MOESM6_ESM.pdf]

删除标识 | 福昕编辑器

SPRINGER NATURE

Corresponding Author Name: Yizhuo Zhang
Manuscript Number: 23-2757

Reporting Summary

Springer Nature wishes to improve the reproducibility of the work that we publish. This checklist is used to ensure good reporting standards and to improve the reproducibility. Please respond completely to all questions relevant to your manuscript. For more information, please read the journal's Guide to Authors.

☑ **Check here to confirm that the following information is available in the Material & Methods section:**

- **The exact sample size (*n*)** for each experimental group/condition, given as a number, not a range
- **A description of the sample collection** allowing the reader to understand whether the samples represent technical or biological replicates (including how many animals, litters, culture, etc.)
- **A statement of how many times the experiment shown was replicated in the laboratory**
- **Definitions of statistical methods and measures**: For small sample sizes (n<5) descriptive statistics are not appropriate, instead plot individual data points
  - Very common tests, such as *t*-test, simple χ^2 tests, Wilcoxon and Mann-Whitney tests, can be unambiguously identified by name only, but more complex techniques should be described in the methods section
  - Are tests one-sided or two-sided?
  - Are there adjustments for multiple comparisons?
  - **Statistical test results**, e.g., ***P* values**
  - Definition of **'center values'** as **median or mean**;
  - Definition of **error bars as s.d. or s.e.m. or c.i.**

Please ensure that the answers to the following questions are reported in the manuscript itself. We encourage you to include a specific subsection in the methods section for statistics, reagents and animal models. Below, provide the page number or section and paragraph number.

Statistics and general methods

Statistics and general methods	Reported in section/paragraph or page #
1. How was the sample size chosen to ensure adequate power to detect a pre-specified effect size? (Give section/paragraph or page #)	For clinical patients, the samples was collected with according inclusion or exclusion criterias.Finally, a total of 57NB patients with complete follow-up data were collected ;Section of Cell lines and clinical samples; page 6 line 93-96;
For animal studies, include a statement about sample size estimate even if no statistical methods were used.	All animal experiments were conducted on 3 to 4-week-old female NCG mice, with 6 mice for each group.<br>Section of Animal experiments; page 10 line 183; 189-194;
2. Describe inclusion/exclusion criteria if samples or animals were excluded from the analysis. Were the criteria pre-established? (Give section/paragraph or page #)	All patients were < 18 years old with initial diagnosis as NB. NB patients >18 years old or with incomplete clinical data were excluded out. These criterias pre-established.<br>Section of Cell lines and clinical samples; page 6, line 93-96;
3. If a method of randomization was used to determine how samples/animals were allocated to experimental groups and processed, describe it. (Give section/paragraph or page #)	Then the mice with tumor were randomly divided in to four groups, with 6 mice for one group.<br>Section of Animal experiments; page 10 line 189-194;
For animal studies, include a statement about randomization even if no randomization was used.	Then the mice with tumor were randomly divided in to four groups, with 6 mice for one group.<br>Section of Animal experiments; page 10 line 189-194;

4. If the investigator was blinded to the group allocation during the experiment and/or when assessing the outcome, state the extent of blinding. (Give section/paragraph or page #)	Investigators were not blinded in this animal experiment. Section of Animal experiments; page 11 line 202-203;
For animal studies, include a statement about blinding even if no blinding was done.	Investigators were not blinded in this animal experiment. Section of Animal experiments; page 11 line 202-203;
5. For every figure, are statistical tests justified as appropriate?	Yes, we have used appropriate methods for statistical tests.
Do the data meet the assumptions of the tests (e.g., normal distribution)?	Yes.
Is there an estimate of variation within each group of data?	Yes.
Is the variance similar between the groups that are being statistically compared? (Give section/paragraph or page #)	Yes. The variance was assumed as similar between compared groups with normal distribution. We have stated this clearly in the section of Statistical analysis; Page 11 line 215-225;

Reagents	Reported in section/paragraph or page #
6. Report the source of antibodies (vendor and catalog number)	Yes; We have described this in Section of Western blotting (WB); Page 7 line 132-138;
7. Identify the source of cell lines and report if they were recently authenticated (e.g., by STR profiling) and tested for mycoplasma contamination	Yes, we have stated these at sections of Cell lines and clinical samples; Page 7 line 86-87;

Animal Models	Reported in section/paragraph or page #
8. Report species, strain, sex and age of animals	Yes, we have stated these at page 10 line 189-194;
9. For experiments involving live vertebrates, include a statement of compliance with ethical regulations and identify the committee(s) approving the experiments.	The design of our experiment does not involve these part;

10. We recommend consulting the ARRIVE guidelines (*PLoS Biol.* **8**(6), e1000412,2010) to ensure that other relevant aspects of animal studies are adequately reported.

Human subjects

Reported in section/paragraph or page #

11. Identify the committee(s) approving the study protocol.	The Ethics Committee of the Sun Yat-sen University Cancer Center;
12. Include a statement confirming that informed consent was obtained from all subjects.	Yes, we have stated these at page 6 line 96-97;
13. For publication of patient photos, include a statement confirming that consent to publish was obtained.	Yes, we have stated these at page 25 line 521;
14. Report the clinical trial registration number (at ClinicalTrials.gov or equivalent).	The design of our experiment does not involve these part;

15. For phase II and III randomized controlled trials, please refer to the CONSORT statement and submit the CONSORT checklist with your submission.

16. For tumor marker prognostic studies, we recommend that you follow the REMARK reporting guidelines.

Data deposition

Reported in section/paragraph or page #

17. Provide accession codes for deposited data. Data deposition in a public repository is mandatory for:<br>a. Protein, DNA and RNA sequences<br>b. Macromolecular structures<br>c. Crystallographic data for small molecules<br>d. Microarray data	Page 25, line 532-534;

Deposition is strongly recommended for many other datasets for which structured public repositories exist; more details on our data policy are available in the Guide to Authors. We encourage the provision of other source data in supplementary information or in unstructured repositories such as Figshare and Dryad. We encourage publication of Data Descriptors (see Scientific Data) to maximize data reuse.

18. If computer code was used to generate results that are central to the paper's conclusions, include a statement in the Methods section under "**Code availability**" to indicate whether and how the code can be accessed. Include version information as necessary and any restrictions on availability.	The design of our experiment does not involve these application.
